# Soil microbial community composition and diversity in the rhizosphere of *Alsophila spinulosa* growing in different habitats within the Chishui *Alsophila* National Nature Reserve in Guizhou Province, China

**DOI:** 10.3389/fmicb.2024.1445255

**Published:** 2024-10-03

**Authors:** Bingjie Che, Weicheng Yang, Qinqin He, Yu Jiang, Bingchen Zhang, Hangdan Chen

**Affiliations:** ^1^School of Life Sciences, Guizhou Normal University, Guiyang, China; ^2^Guizhou Chishui Alsophila National Nature Reserve Administration Bureau, Chishui, China

**Keywords:** relict plant, rhizosphere soil, microbial community structure, plant protection, high-throughput sequencing

## Abstract

The rhizosphere is considered a highly complex and dynamic ecosystem. Rhizosphere soil microorganisms influence the growth and development of plants by mediating the transformation and absorption of nutrients. In order to explore the microbial community composition and diversity of *Alsophila spinulosa* growing in different habitats. Rhizosphere samples were collected from four different habitats within the Chishui *Alsophila* National Nature Reserve in Guizhou Province, China. According to the high-throughput sequencing results of 16 s rDNA and ITS, Proteobacteria and Ascomycota were the most abundant bacterial and fungal phyla in the rhizosphere soil of all four habitats. The alpha diversity analysis indicated that two particular habitats, Buddha Rock and Botanical Garden, harbored the highest microbial richness and diversity. LEfSe analysis revealed that Buddha Rock contained the highest relative abundance of Bacteroidetes compared to the other three study areas. Meanwhile, Tiantang Gou contained the highest relative abundance of Basidiomycota. Bacterial community composition and diversity were greatly influenced by soil pH, while fungal community composition and diversity were greatly influenced by available phosphorus, organic carbon, sucrase, and urease. The results of this study provide a scientific basis for the habitat restoration of *A. spinulosa*, and the improvement of the structure of the *A. spinulosa* rhizosphere soil microbial community. Laying a theoretical foundation for the next screening of inter-root functional flora.

## 1 Introduction

The rhizosphere system is formed by interactions among plants, soil, and microorganisms (Hashidoko, 2005; Albareda et al., 2006), and acts as a hub for the storage and cycling of chemical nutrients (Aly et al., 2011; Yang et al., 2017). As the most active biological component of soil ecosystems, soil microorganisms promote material transformation, energy flow, and biogeochemical cycling (Bridge and Spooner, 2021). Rhizosphere soil microorganisms are the source of the plant microbiome and are responsible for driving nutrient cycling, including promoting the decomposition of organic matter in the soil (Jacoby et al., 2017), the absorption and transformation of nutrients by plant roots, and the growth and development of plants (Berg and Smalla, 2009; Philippot et al., 2013). For example, microorganisms support plant growth and development by promoting the utilization of nitrogen, phosphorus, and water (Yang et al., 2017).

The plant root microbiome is crucial for plant and ecosystem health (Berg and Smalla, 2009; Philippot et al., 2013). Meanwhile, compounds released by plants can directly affect the rhizosphere soil and associated microorganisms. Rhizosphere microorganisms are influenced by soil type (Berg and Smalla, 2009), environmental conditions (Rasche et al., 2006), and plant genotype (Raaijmakers et al., 2009). In addition, rhizosphere microorganisms produce specific “rhizosphere effects” in different habitats to enhance plant adaptability to adverse environmental conditions. On the other hand, plant roots influence nutrient selection and enrichment, thereby increasing rhizosphere microbial diversity (Lynch and Whipps, 1990). These effects are particularly important in environments characterized by high salinity, drought, and water scarcity (Krasensky and Jonak, 2012). Overall, while plants are affected by the large number of interconnected microorganisms which make up the rhizosphere community, the rhizosphere is also affected by plants (Philippot et al., 2013; Rajput et al., 2021).

Studying the rhizosphere microbiomes of plants in different habitats can reveal the relationship between plants and microorganisms, as well as the adaptability of the soil–plant-microbe system to stressful environmental conditions. Of particular interest is the “living fossil” and Mesozoic tree fern *Alsophila spinulosa* (Korall et al., 2007), which can help illuminate ancient climatic and environmental changes. *A. spinulosa* also has ornamental and medicinal value (Cheng et al., 1990; Chiang et al., 1994; Sureshkumar et al., 2018), and holds great potential for development. *A. spinulosa* specimens growing in the Chishui *Alsophila* National Nature Reserve in Guizhou Province, China, have been observed to exhibit self-reproductive difficulties, and suffer herbivory by *Phthonoloba viridifasciata* and *Rhoptroceros cyatheae*. Despite its protected status, there has been little research into the rhizosphere community associated with *A. spinulosa*, and the microbial diversity and community structure of the *A. spinulosa* root microbiome remain uncharacterized. Here, we studied the composition and diversity of soil microbial communities associated with *A. spinulosa* growing in different habitats within the Chishui *Alsophila* National Nature Reserve. Our results provide a reference for the continued protection of *A. spinulosa*, and may be helpful in the establishment of restoration areas to achieve protection goals. These findings also lay a foundation for further research on the functions of *A. spinulosa* rhizosphere microorganisms.

## 2 Materials and methods

### 2.1 Overview of the research area

**Figure 1:**
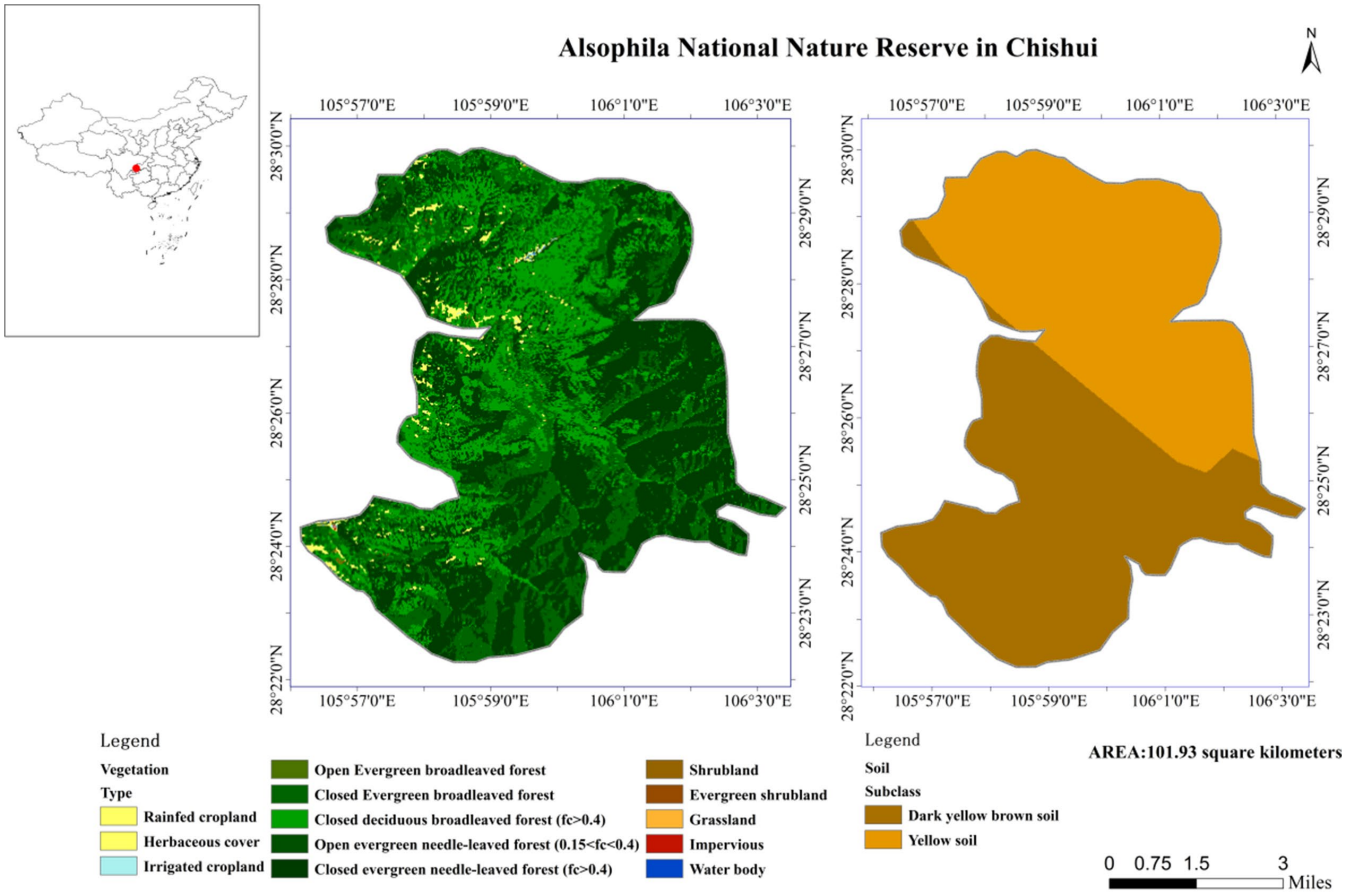
The research site in the study.

This research was approved by the *Alsophila* Management Bureau of Chishui City, Guizhou Province, China. The study site was located in Chishui City, Guizhou Province, southwestern China. The Chishui *Alsophila* National Nature Reserve (longitude 105°41′-106°58′E, latitude 28°20′-28°35′N) is a closed terrain characterized by steep terrain and deep river valleys, and contains Danxia landform features as well as varied environmental and climatic conditions. Certain sections of the protected area have extremely high oxygen concentrations and high environmental humidities. The protected area belongs to a subtropical humid monsoon climate, with an average annual temperature of 17.7°C, an average annual precipitation of 1,200–1,300 mm, and a relative humidity of 90%. *A. spinulosa* specifically grows on sandy soil with pH 4.5–5.5 (Figure 1).

### 2.2 Site selection and soil collection

**Figure 2:**
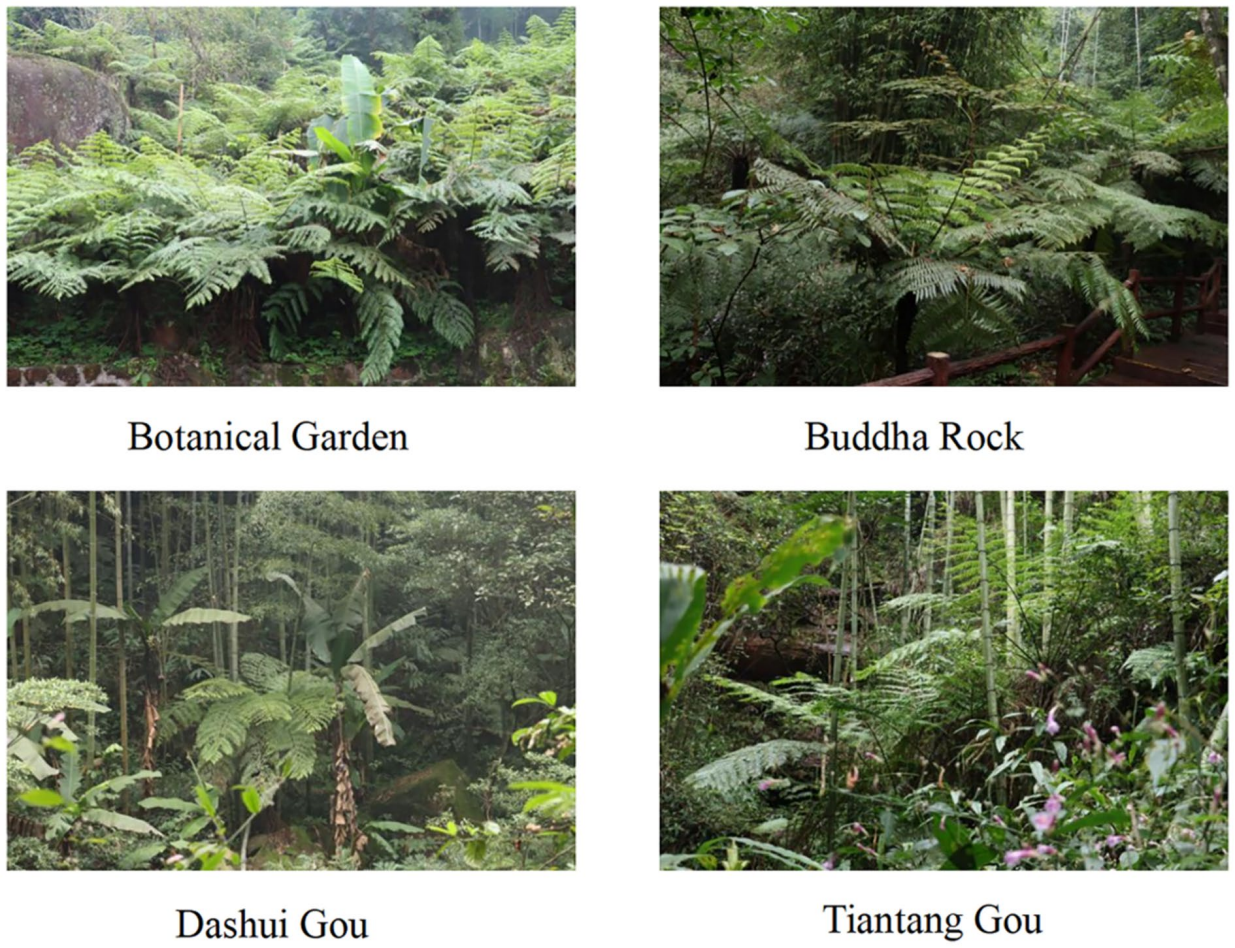
Photographs of four habitats within the Chishui *Alsophila spinulosa* Nature Reserve.

Four experimental areas representing different habitats were selected within the protected area (Figure 2): Botanical Garden (established in 2014 as a garden for artificially-cultivated *A. spinulosa*), Buddha Rock (developed as a tourist attraction), Dashui Gou (restored after human activities), and Tiantang Gou (pristine habitats undisturbed by human activities and animal agriculture), sampling at four sites with different levels and modes of interference. The surface soil was removed from three randomly-selected healthy plants within each experimental area. The rhizosphere soil was collected by shaking, and roots with similar growth conditions were selected as five replicates. Tweezers were used to remove impurities from the soil, which was immediately placed in sterile bags. All soil samples were collected in sterile plastic bags and immediately transported to the laboratory on dry ice. According to research needs, the soil samples were divided into at least two sub-samples: one was stored at −80°C for high-throughput DNA sequencing analysis and the other was air-dried for physicochemical index detection and analysis. The method described by Bao (2000) was used to determine soil chemical properties, including soil organic matter (SOM), organic carbon (OC), total nitrogen (TN), available nitrogen (AN), available phosphorus (AP), pH, sucrase, and urease.

### 2.3 DNA extraction, PCR amplification, and Illumina MiSeq sequencing

E.Z.N.A. soil DNA kits (Omega Biotek, Norcross, GA, United States) were used to extract DNA from soil samples, according to the manufacturer’s instructions. Next, 1% agarose gel electrophoresis was utilized to evaluate the quality of the DNA, and a NanoDrop 2000 spectrophotometer (ThermoFisher Scientific, Waltham, MA, United States) was used to determine the concentration and purity of the DNA.

The extracted DNA samples were used to conduct a microbial community analysis via polymerase chain reaction (PCR). Bacteria were identified using the V3-V4 region of the 16S rDNA gene, including 338F (5′-ACTCCTACGGGGCAGCAG-3′) and 806R (5′-GACTACHVGGGTWTCTAAT-3′), and fungi were identified using ITS1F (5′-CTTGGTCATTTAGAGGAGAGTAA-3′) and ITS2R (5′-GCTGGTTCTTCGATGC-3′). PCR was performed in a 20-μL reaction system (TransStart FastPfu DNA Polymerase), including 4 μL of 5x TransStart FastPfu buffer, 2 μL of 2.5 mM dNTPs, 0.8 μL each of upstream and downstream primers (5 μM), 0.4 μL of TransStart FastPfu DNA polymerase, and 10 ng of template DNA. PCR was performed on an ABI GeneAmp 9700 (ThermoFisher Scientific, Waltham, MA, United States) according to the following procedure: pre-denaturation at 95°C for 3 min, 27 cycles of denaturation at 95°C for 30 s, annealing at 55°C for 30 s, and extension at 72°C for 30 s, followed by stable extension at 72°C for 10 min, and storage at 4°C. PCR products were recovered on 2% agarose gels and purified using the PCR Clean-Up Kit (Yuhua, Shanghai, China).

The extracted DNA was sent to Shanghai Meiji Biomedical Technology Co., Ltd. for sequencing on an Illumina MiSeq PE300 platform. The raw data were uploaded to the NCBI SRA database. Fastp ver. 0.19.6 (Magoč and Salzberg, 2011)^1^ was used to perform quality control on the original double ended sequences, and FLASH ver. 1.2.11 (Chen et al., 2018)^2^ was used to perform splicing (Magoč and Salzberg, 2011). Then the optimized sequences were clustered into operational taxonomic units (OTUs) using UPARSE (version 7.1) (Edgar, 2013) with a 97% sequence similarity level. Representative microbial sequences were compared and annotated using the Silva database (Release 138^3^) (Quast et al., 2013), and chloroplast and mitochondrial sequences were removed. Comparison and annotation of representative fungal sequences using the Unite database (Unite, Rlease 8.0^4^) (Kõljalg et al., 2020). The obtained OTUs were homogenized according to the minimum sample sequence method, and the minimum reads for the bacterial and fungal sequences were 19,604 and 15,876, respectively. The subsequent diversity analyses were based on the standardized abundances of the OTUs.

### 2.4 Data processing

Data analysis was conducted on the Meiji Bio Cloud platform.^5^ In order to determine statistically significant differences among soil chemical properties, a one-way analysis of variance (ANOVA) followed by Tukey’s test were conducted using R4.2.1 (R Core Team, 2022)^6^. RDP Classifier Brayesian (Wang et al., 2007) algorithm was used to classify and analyze representative OTUs. The species composition and relative abundance of each sample were calculated and used to generate bar charts and Venn diagrams. The alpha (*α*) diversity included the ACE, Shannon-Wiener, Simpson, and Chao indices of the soil bacterial and fungal communities was estimated at the OTU levels using MOTHUR (Schloss et al., 2009) and Unifrac (Lozupone and Knight, 2005), respectively. QIIME (Caporaso et al., 2010) was used to calculate the beta (*Β*) difference distance matrix for systematic clustering analysis. The Kruskal–Wallis test was used to analyze the α-diversity index of bacteria and fungi in the samples. The Wilcoxon signed-rank test and *T*-test (false discovery rate correction) were used for pairwise comparison. The principal coordinates analysis (PCoA) of *β*-diversity was calculated based on the Bray–Curtis algorithm using R (version 3.3.1) (R Core Team, 2022), the model of difference test between groups was an Adonis, and the number of replacements was 999. Linear discriminant analysis Effect Size (LEfSe) analysis (Segata et al., 2011) was used to determine species with significant differences among different plots (phylum to genus) (LDA > 4, *p* < 0.05). The vegan package (vsesion2.4.3)^7^ in R was used to generate a box plot and principal component analysis (RDA) community heat map.

## 3 Results

### 3.1 Physical and chemical properties of *Alsophila spinulosa* rhizosphere soil

Significant differences were observed in the physicochemical properties of the *A. spinulosa* rhizosphere soil among four different habitats (Table 1). All tested soils were weakly acidic (pH: 4.55–6.62), with Buddha Rock having the highest pH (*p* < 0.05). The contents of SOM, OC, TN, and AN in the rhizosphere soil at Tiantang Gou were significantly higher (*p* < 0.05) than those of the other three plots. The AP content was highest in the rhizosphere soil at Buddha Rock and lowest in the rhizosphere soil at Tiantang Gou. The contents of sucrase and urease were highest in the rhizosphere soil at Tiantang Gou, and no significant differences were observed in the contents of these enzymes between Dashui Gou and Buddha Rock. With the exception of AP, the contents of all other physicochemical indicators were lowest in the rhizosphere soil at the Botanical Garden (*p* < 0.05).

**Table 1:** Physical and chemical indicators of *A. spinulosa* rhizosphere soil among four habitats.

Physicochemical indicator	Tiantang Gou	Dashui Gou	Buddha Rock	Botanical Garden
pH	5.24 ± 0.00c	4.55 ± 0.0.5d	6.62 ± 0.03a	5.85 ± 0.01b
SOM (g/kg)	24.56 ± 0.21a	22.15 ± 0.15c	22.64 ± 0.29b	9.17 ± 0.08d
OC (g/kg)	14.25 ± 0.13a	12.85 ± 0.09c	13.14 ± 0.17b	5.32 ± 0.05d
TN (g/kg)	1.46 ± 0.04a	1.36 ± 0.05b	1.23 ± 0.02c	0.84 ± 0.01d
AN (mg/kg)	155.23 ± 1.04a	133.74 ± 1.43c	143.09 ± 1.71b	63.49 ± 1.88d
AP (mg/kg)	2.07 ± 0.06d	3.50 ± 0.10c	23.43 ± 0.15a	3.80 ± 0.10b
Sucrase (mg/g)	4.55 ± 0.24a	3.75 ± 0.04b	3.70 ± 0.09b	2.02 ± 0.05c
Urease (μg/g)	396.59 ± 27.19a	275.26 ± 13.84b	275.57 ± 16.89b	175.14 ± 4.45c

Different lowercase letters indicate statistically significant differences among habitats.

### 3.2 Differences in *Alsophila spinulosa* rhizosphere soil microbial communities among four different habitats

#### 3.2.1 Composition and diversity of bacterial communities in *Alsophila spinulosa* rhizosphere soil

**Figure 3:**
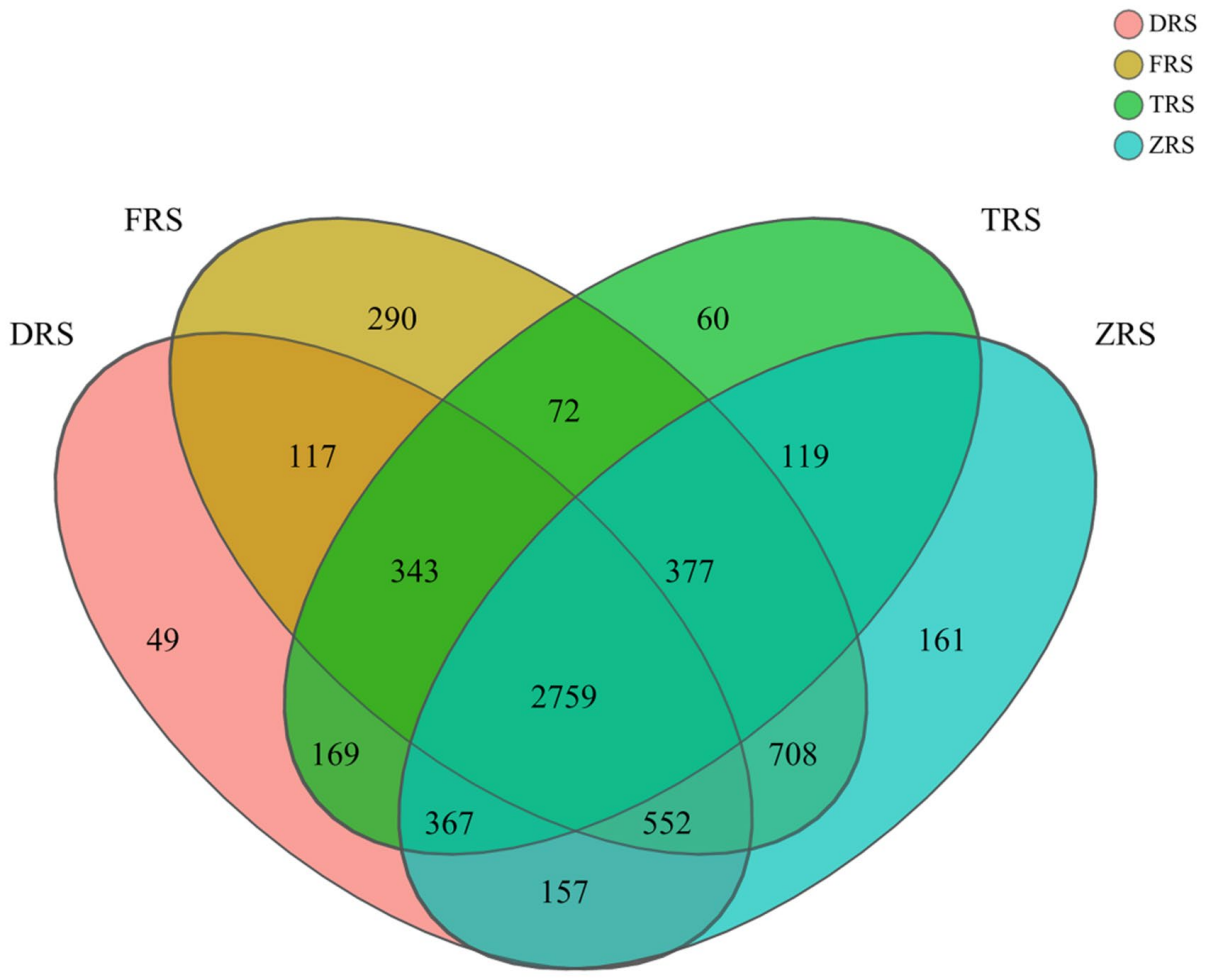
Venn diagram of soil bacteria identified in the *A. spinulosa* rhizosphere soils among four habitats. DRS, FRS, TRS, and ZRS represent the inter-root soils in Dashui Gou, Buddha Rock, Tiantang Gou, and the Botanical Garden, respectively.

A total of 6,300 bacterial OTUs were annotated among the tested soil samples, representing 35 phyla, 112 classes, 261 orders, 401 families, 71 genera, and 1,647 species. The *A. spinulosa* rhizosphere microbial community composition was found to differ among habitats. A total of 4,513, 5,218, 4,266, and 5,200 OTUs were annotated in inter-root soil samples from Dashui Gou (DRS), Buddha Rock (FRS), Tiantang Gou (TRS), and Botanical Garden (ZRS), respectively. A total of 2,759 core OTUs (found at all sample) were annotated among all samples, accounting for 43.79% of all OTUs. Dashui Gou, Buddha Rock, Tiantang Gou, and the Botanical Garden were found to harbor 49, 290, 60, and 161 distinct OTUs, respectively (Figure 3).

**Figure 4:**
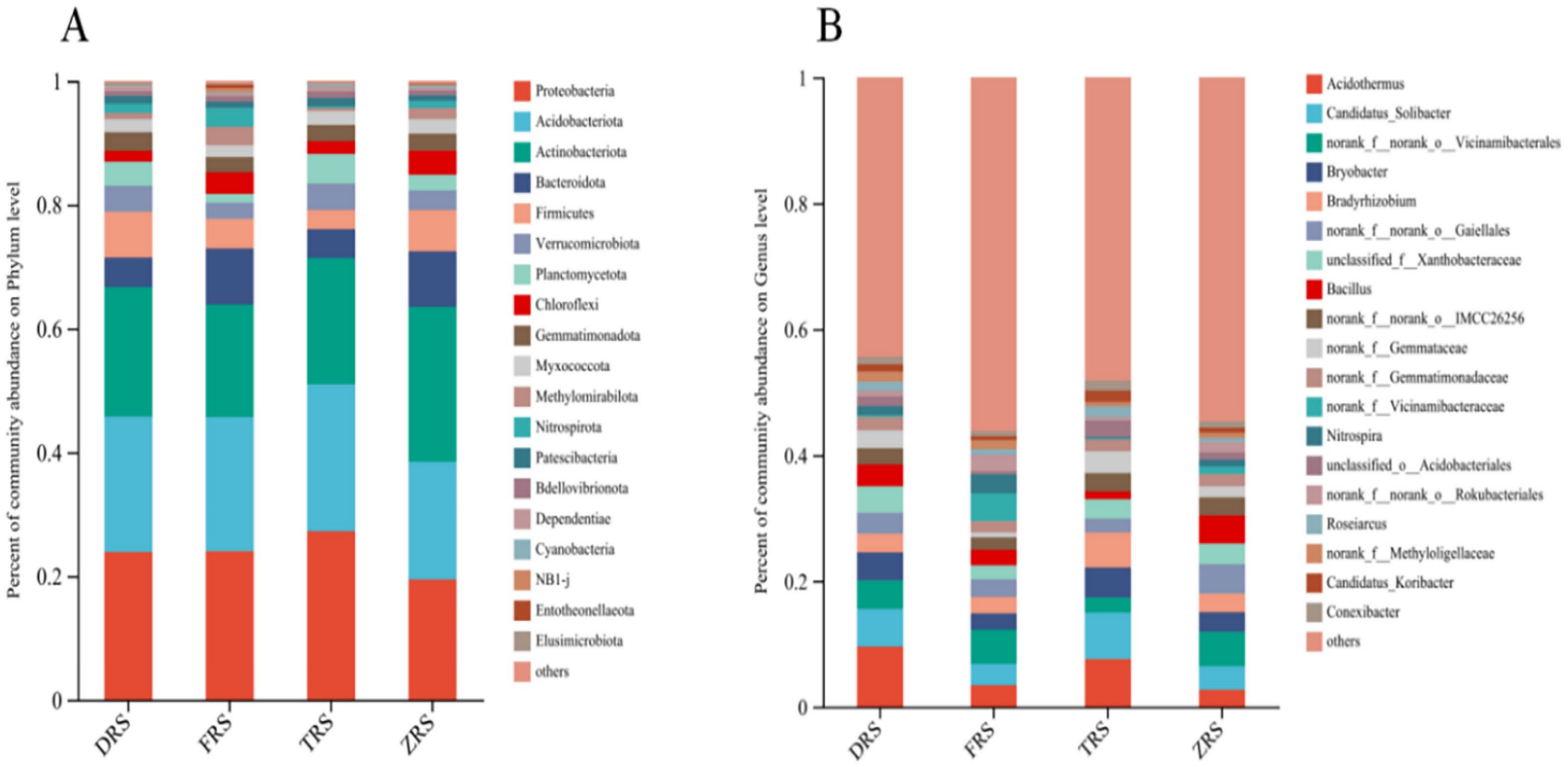
Relative abundances of bacterial phyla **(A)** and genera **(B)** identified in the *A. spinulosa* rhizosphere soils among four habitats.

Significant differences were observed in the abundances of bacterial groups in the *A. spinulosa* rhizosphere soil among different habitats. The main bacterial phyla in Tiantang Gou were Proteobacteria (27.24%), Acidobacteriota (23.69%), Actinobacteriota (13.30%), Planctomycota (4.78%), and Verrucomicrobiota (4.30%). The main bacterial phyla in Dashui Gou were Proteobacteria (23.89%), Acidobacteriota (21.88%), Actinbacteriota (20.95%), Firmicutes (7.41%), and Verrucomimicrobiota (4.20%). The main bacterial phyla in Buddha Rock were Proteobacteria (24.02%), Acidobacteriota (21.62%), Actinbacteriota (18.26%), Bacteroidota (9.04%), and Firmicutes (4.79%). The main bacterial phyla in the Botanical Garden were Actinobacteriota (25.06%), Proteobacteria (19.45%), Acidobacteriota (18.99%), Bacteroidota (8.96%), and Firmicutes (6.68%). The main bacterial genera in Tiantang Gou were *Acidothermus* (7.59%), Candidatus_Solibacte (7.40%), *Bradyrhizobium* (5.58%), *Bryobater* (4.76%), and OTUs from families Gemmataceae and Xanthobacteriaceae (3.14%), unclassified_f_Xanthobacteriaceae (3.12%), and norank_f_norank_o_IMCC26256 (2.93%). The main bacterial genera in Dashui Gou were *Acidothermus* (9.56%), Candidatus_Soliactor (5.98%), OTUs from families Vicinamidobacteriales (4.55%), *Bryobacter* (4.45%), unclassified_f_Xanthobacteriaceae (4.19%), OTUs from families Gaiellales (3.33%), and *Bacillus* (3.15%). The main bacterial genera in Buddha Rock were *Acidothermus* (3.45%), Candidatus_Soliactor (3.39%), OTUs from families Vicinamidobacteriales (5.41%), *Nitrospira* (2.97%), *Bradyrhizobium* (2.67%), OTUs from families Gaiellales (2.75%), and OTUs from families Vicinamidobacteriaceae (4.44%). The main bacterial genera in the Botanical Garden were *Candidatus_Solibater* (3.73%), OTUs from families Vicinamidobacteriale (5.48%), *Bryobacter* (3.11%), *Bradyrhizobium* (2.98%), OTUs from families Gaiellales (4.64%), unclassified_f_Xanthobacteriaceae (3.28%), and *Bacillus* (4.46%) (Figure 4).

**Figure 5:**
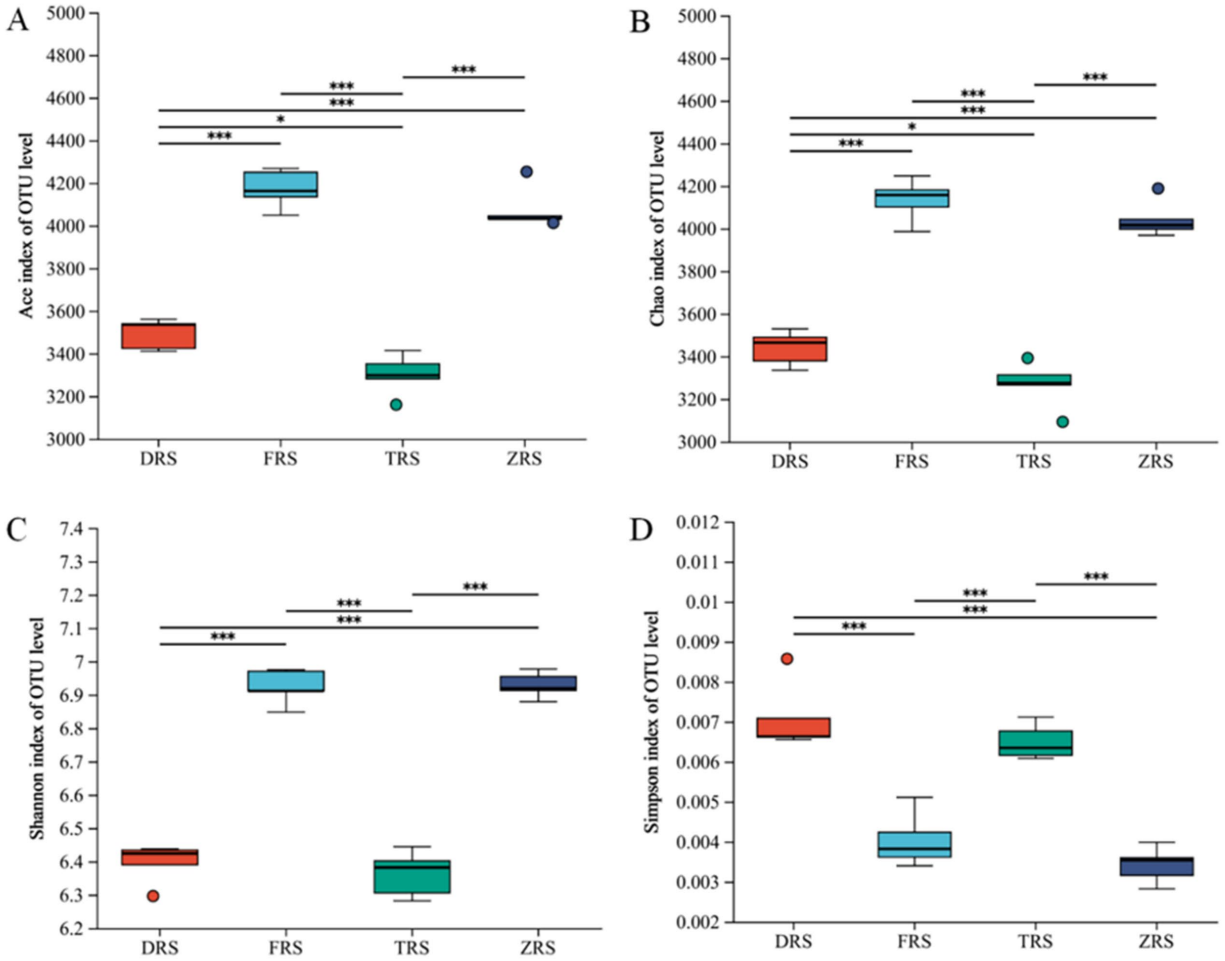
Box plots of bacterial alpha (*α*) diversity in the *A. spinulosa* rhizosphere soils among four habitats: the ACE index **(A)**; the Chao index **(B)**; the Shannon index **(C)**; the Simpson index **(D)**.

According to the *α* diversity index analysis, the ACE and Chao1 indices of community richness were highest in Buddha Rock, followed by the Botanical Garden, Dashui Gou, and Tiantang Gou. The ACE and Chao1 indices were significantly higher in Buddha Rock than in Dashui Gou and Tiantang Gou, with significant differences observed between the groups. The Shannon—Wiener and Simpson indices of community diversity were highest in Buddha Rock and the Botanical Garden, which were significantly higher than those in Dashui Gou and Tiantang Gou (Figure 5).

#### 3.2.2 Composition and diversity of fungal communities in *Alsophila spinulosa* rhizosphere soil

**Figure 6:**
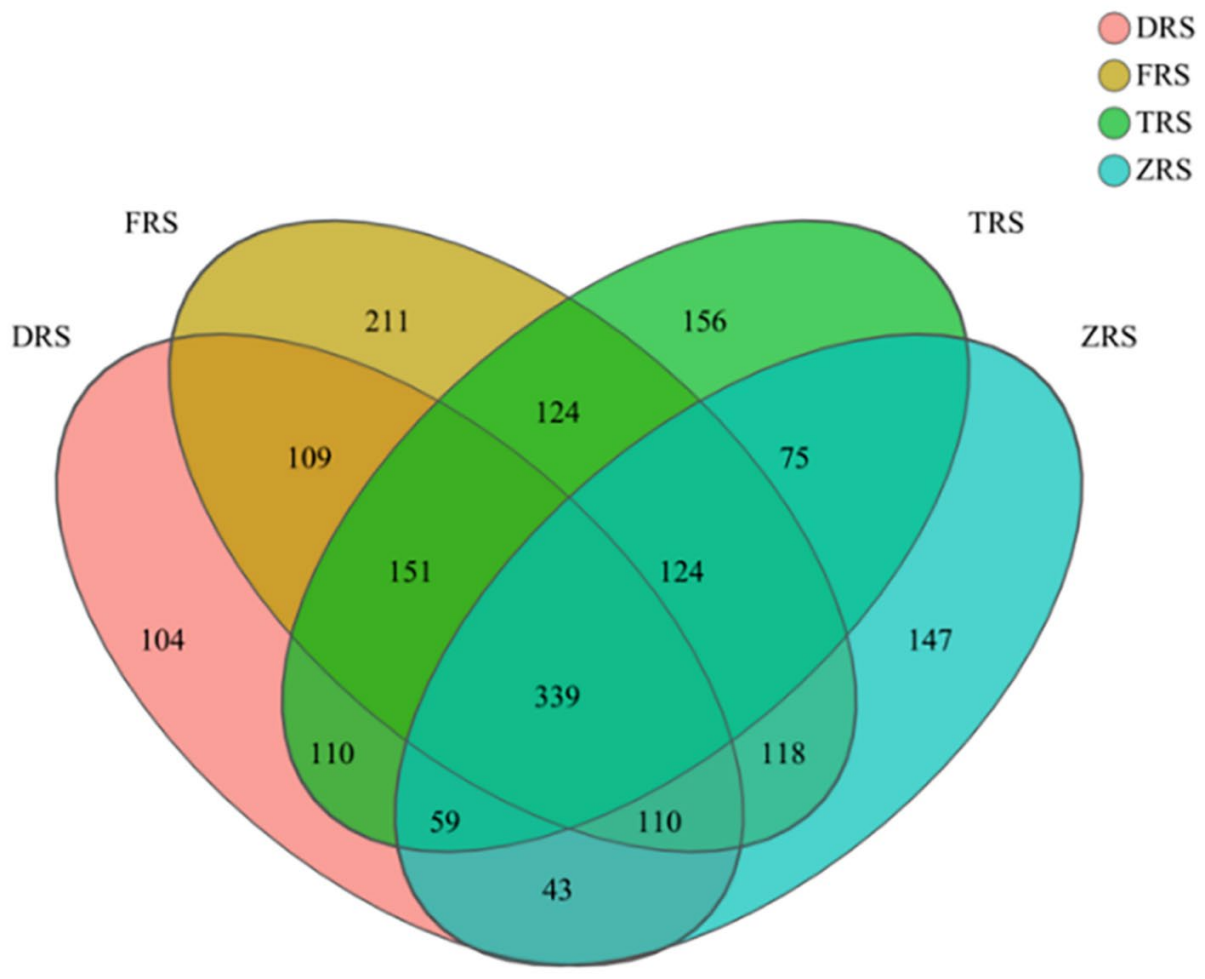
Venn diagram of soil fungi identified in the *A. spinulosa* rhizosphere soils among four habitats. DRS, FRS, TRS, and ZRS represent the inter-root soils in Dashui Gou, Buddha Rock, Tiantang Gou, and the Botanical Garden, respectively.

A total of 1,868 fungal OTUs were annotated among the tested soil samples, representing 48 classes, 14 phyla, 111 orders, 237 families, 470 genera, and 748 species. A total of 1,025, 1,286, 1,138, and 1,015 OTUs were identified in the DRS, FRS, TRS, and ZRS soil samples, respectively. A total of 339 core OTUs were identified among samples, accounting for 29.79% of all OTUs. In all, DRS, FRS, TRS, and ZRS contained 104, 211, 156, and 147 distinct OTUs, respectively (Figure 6).

**Figure 7:**
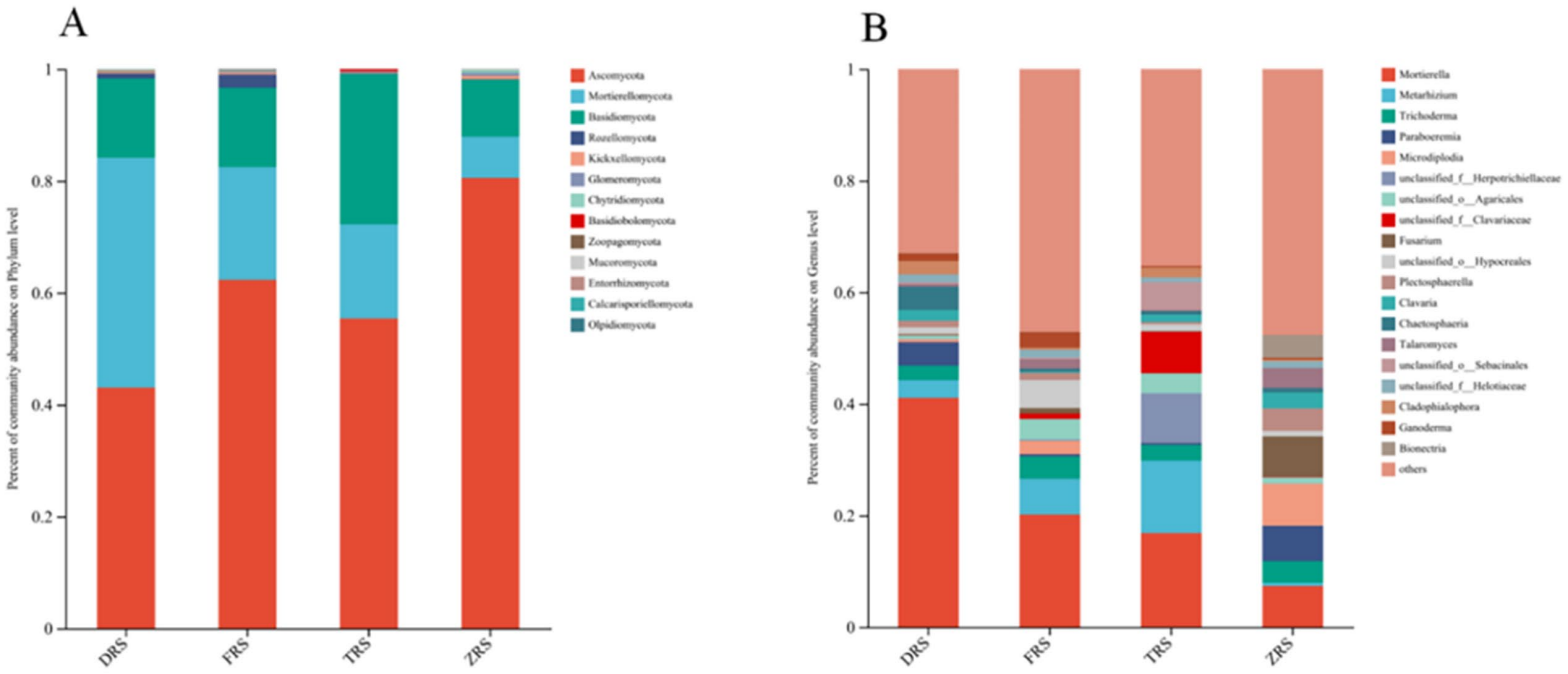
Relative abundances of **(A)** fungal phyla and **(B)** genera (B) identified in the *A. spinulosa* rhizosphere soils among four habitats.

The dominant fungal phyla were the same among all four habitats, and included Ascomycota (54.81%), Basidiomycota (21.22%), Morterellomycota (15.93%), and Rozellomycota (6.69%) (Figure 6). The main fungal genera in Tiantang Gou were *Mortierella* (16.84%), *Metarhizium* (12.97%), *Trichoderma* (2.82%), unclassified_f_Herotrichiellaceae (8.86%), unclassified_o_Aggregates (3.57%), and unclassified_f_Clavariaceae (7.43%). The main fungal genera in Dashui Gou were *Mortierella* (41.06%), *Metarhizium* (3.17%), *Chaetosphaeria* (4.16%), *Trichoderma* (2.60%), and *Paraboeremia* (4.21%). The main fungal genera in Buddha Rock were *Mortierella* (20.13%), *Metarhizium* (6.42%), *Trichoderma* (4.02%), unclassified_o_Agarics (3.73%), and *Ganoderma* (2.80%). The main fungal genera in the Botanical Garden were *Mortierella* (7.36%), *Trichoderma* (3.89%), *Paraboeremia* (6.35%), *Micropodia* (7.53%), *Fusarium* (7.22%), *Bionectria* (4.09%), and *Talaromyces* (3.56%) (Figure 7).

**Figure 8:**
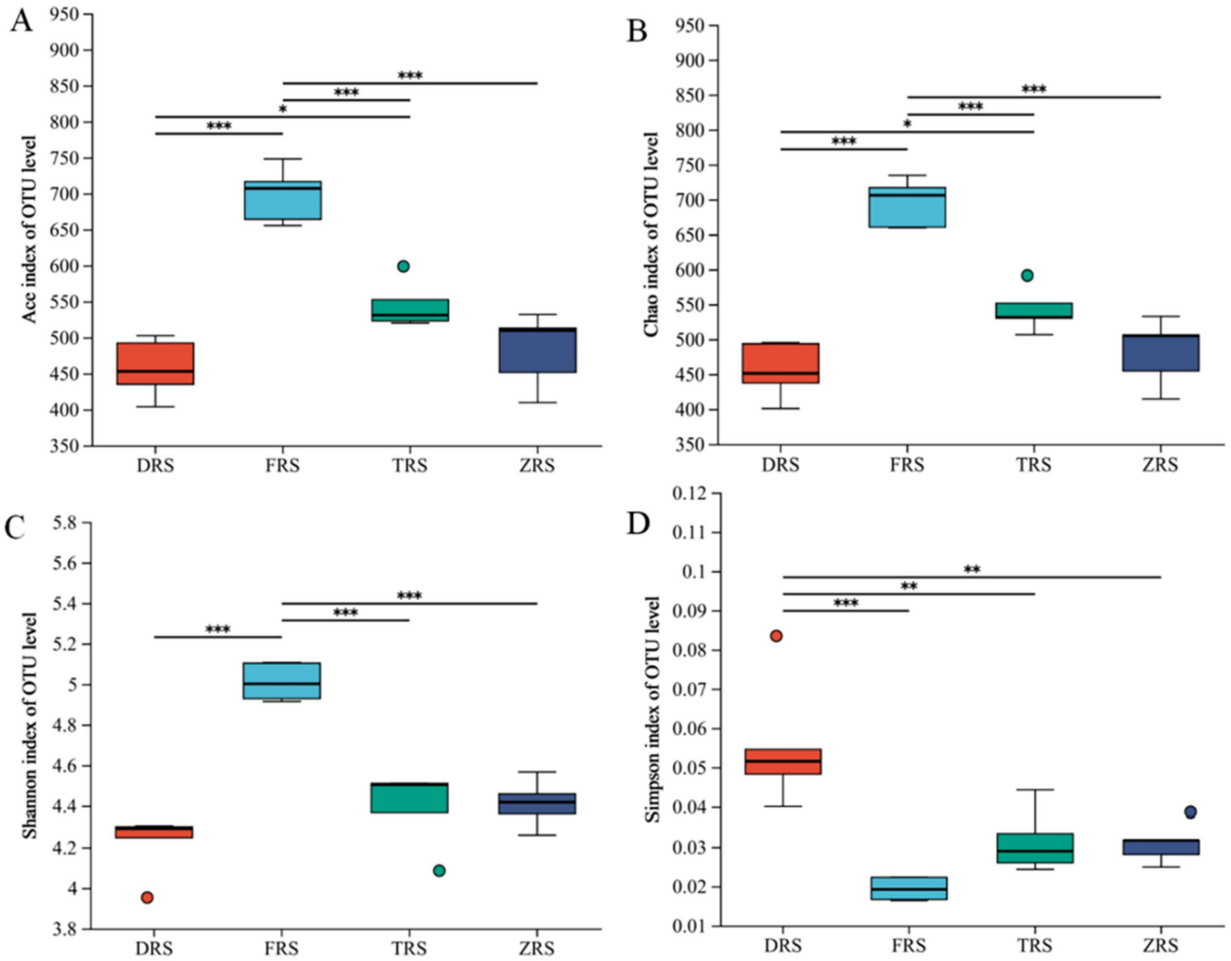
Box plots of fungal alpha (α) diversity in the *A. spinulosa* rhizosphere soils among four habitats: **(A)** the ACE index; **(B)** the Chao index; **(C)** the Shannon index; **(D)** the Simpson index.

According to the *α* diversity index analysis, the ACE and Chao1 indices of community richness were significantly higher in Buddha Rock than in Dashui Gou and Tiantang Gou, with significant differences observed between the groups. The Shannon—Wiener and Simpson indices of community diversity were highest in Buddha Rock and the Botanical Garden, which were significantly higher than those in Dashui Gou and Tiantang Gou (Figure 8).

### 3.3 Differences in *Alsophila spinulosa* rhizosphere soil microbial community structure among four different habitats

#### 3.3.1 Differences in bacterial community structure among *Alsophila spinulosa* rhizosphere soil samples

**Figure 9:**
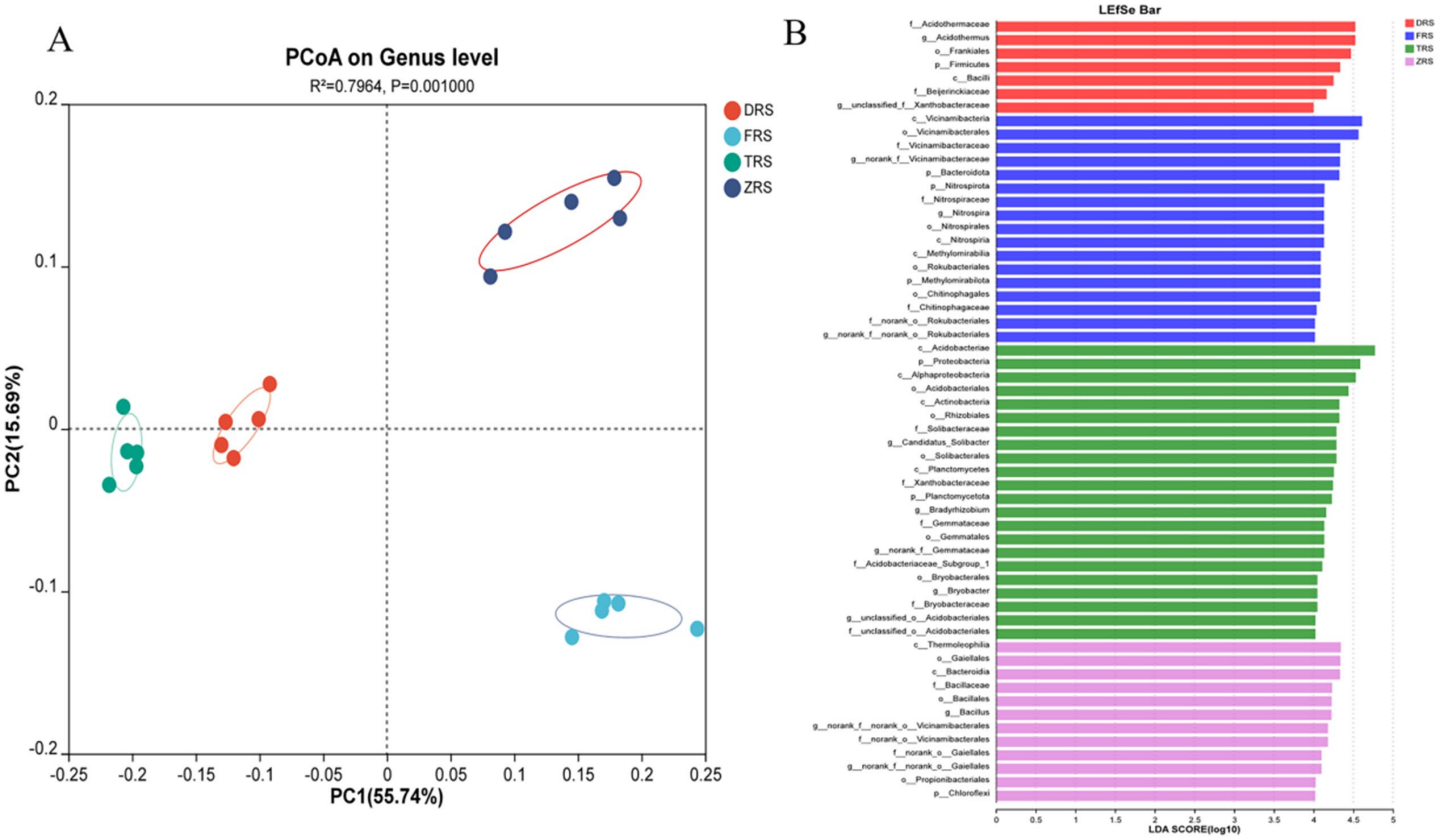
**(A)** PCoA based on the Bray-Curtis distance algorithm was used to analyze the genus-level bacterial community structure of the *A. spinulosa* rhizosphere soils among different habitats. **(B)** LEfSe analysis of the *A. spinulosa* rhizosphere soils among different habitats (significant differences were determined at LDA > 4).

PCoA based on the Bray-Curtis distance algorithm was used to analyze the genus-level bacterial community structure of the *A. spinulosa* rhizosphere soils among different habitats. The bacterial communities among different habitats showed good separation and significant differences. Specifically, Tiantang Gou was clearly separated from Dashui Gou, Buddha Rock, and the Botanical Garden. PC1 and PC2 are the two most prominent characteristics leading to differences among samples, and were found to explain 71.43% of the variation among bacterial communities (Figure 9A). Figure 9B reflects the results of LEfSe and displays potential bacterial biomarkers in the rhizosphere soil at each sampling point. A total of nine bacterial species were identified at the phylogenetic level, excluding unidentified species. According to the LDA results, the main bacterial phylum in Dashui Gou was Firmicutes; those in Buddha Rock were Bacteroidota, Nitrospira, and Methylmicrobiota; and those in Tiantang Gou were Proteobacteria, Planctomycota, and Chloroflexi.

#### 3.3.2 Differences in fungal community structure among *Alsophila spinulosa* rhizosphere soil samples

**Figure 10:**
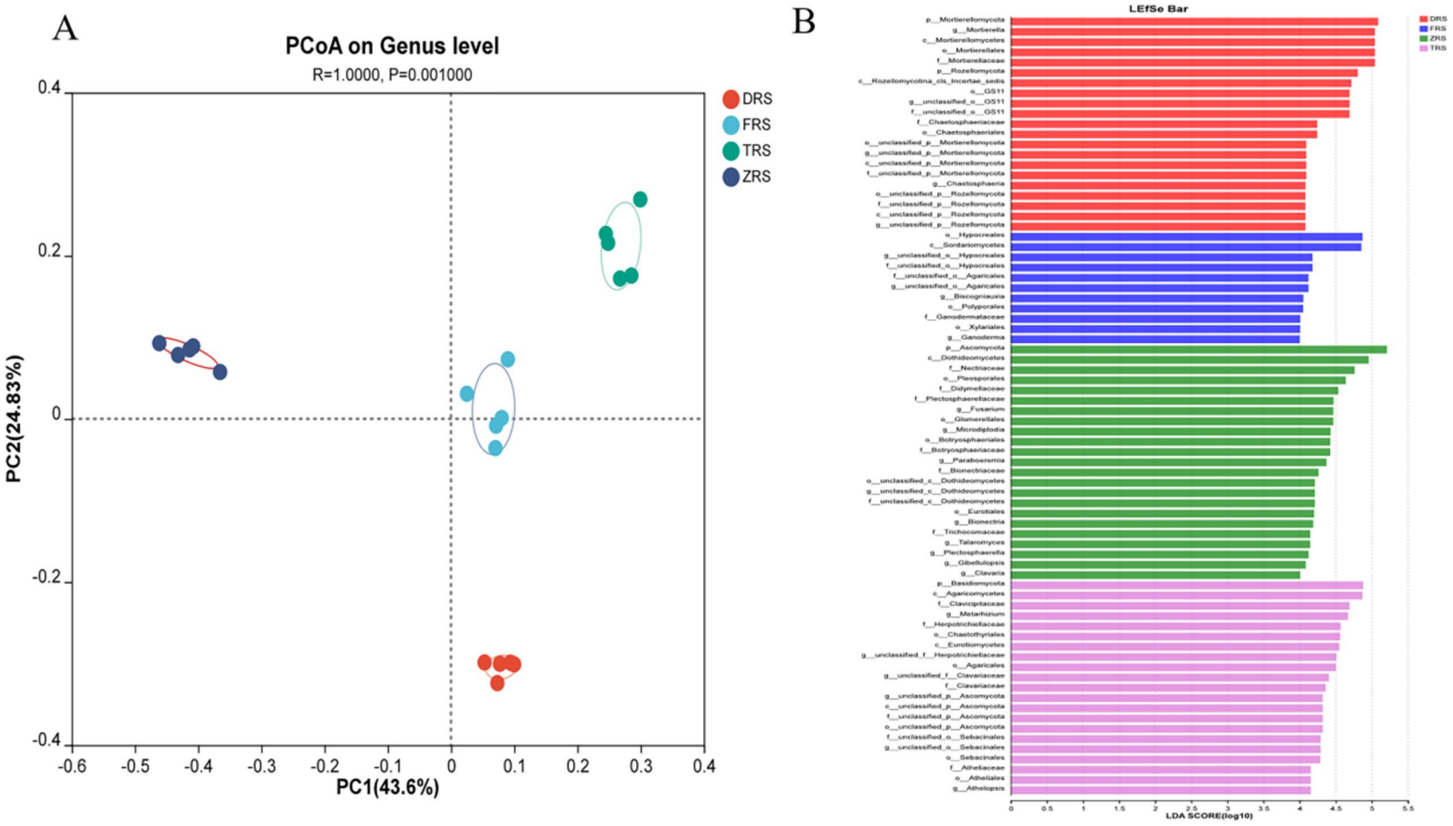
**(A)** PCoA based on the Bray-Curtis distance algorithm was used to analyze the genus-level fungal community structure of the *A. spinulosa* rhizosphere soils among different habitats. **(B)** LEfSe analysis of the *A. spinulosa* rhizosphere soils among different habitats (significant differences were determined at LDA > 4).

PCoA based on the Bray-Curtis distance algorithm was used to analyze the genus-level fungal community structure of the *A. spinulosa* rhizosphere soils among different habitats. The fungal communities among different habitats showed good separation and significant differences. Specifically, Tiantang Gou was clearly separated from Dashui Gou, Buddha Rock, and the Botanical Garden. PC1 and PC2 are the two most prominent characteristics leading to differences among samples, and were found to explain 71.43% of the variation among fungal communities (Figure 10A). According to the LDA results (Figure 10B), the main fungal phylum in Tiantang Gou was Ascomycota; that in the Botanical Garden was Basidiomycota; and those in Dashui Gou were Mortierellomycota and Rozellomycota.

### 3.4 Correlations between rhizosphere soil physicochemical properties and microbial communities

**Figure 11:**
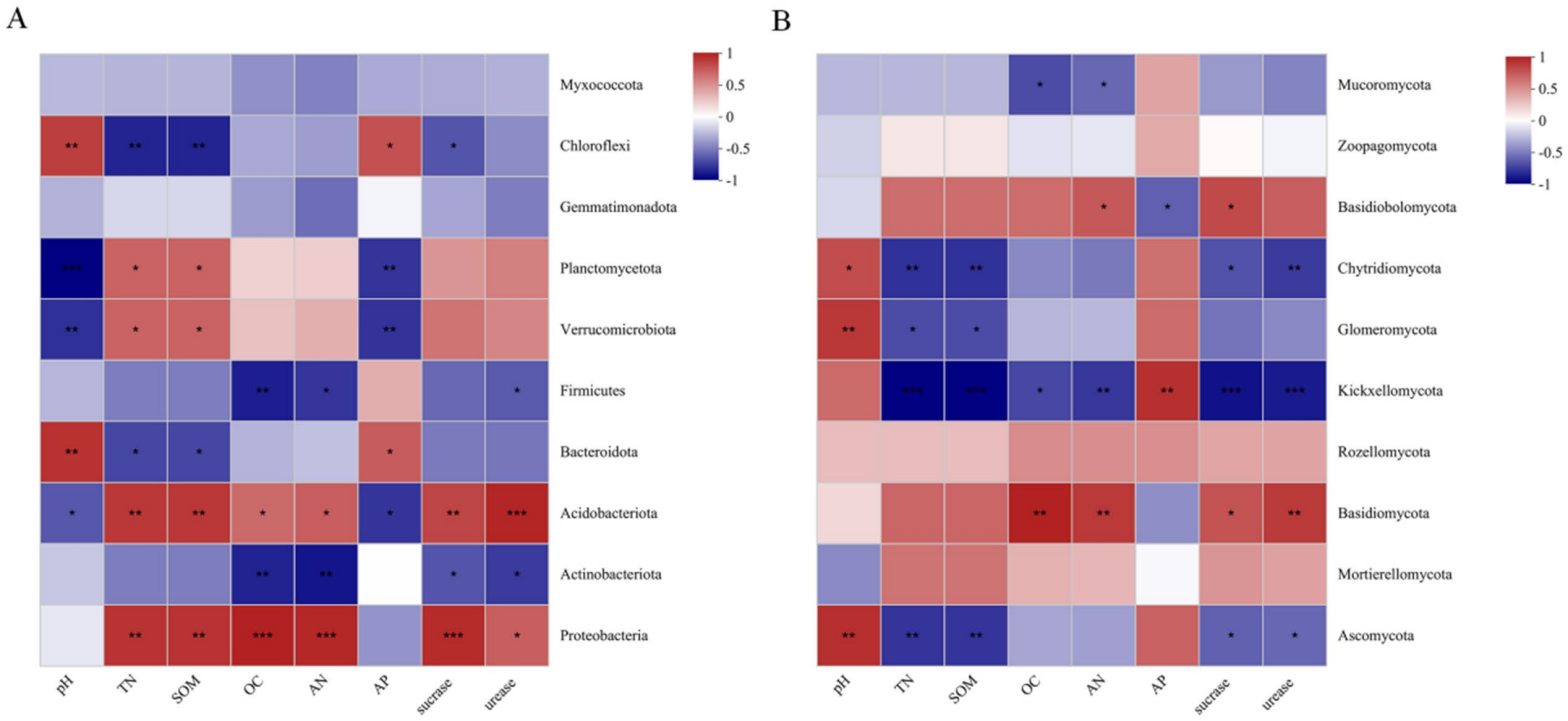
RDA/CCA analysis of soil physicochemical indicators and bacterial **(A)** and fungal **(B)** diversity among different habitats.

RDA/CCA analysis was performed to evaluate the relationships between soil physicochemical properties and microbial communities (Figure 11). The distance between Buddha Rock and Botanical Garden samples was relatively close, indicating that the bacterial community structures among these two habitats were relatively similar. Soil pH was found to have a relatively strong impact on the bacterial community in the Botanical Garden. According to the correlation analysis between rhizosphere soil fungal communities and physicochemical indicators, the horizontal and vertical axes explained 26.48 and 21.33% of the total variability, respectively. In Dashui Gou, AP, pH, and OC, among other factors, strongly influenced the fungal community structure.

**Figure 12:**
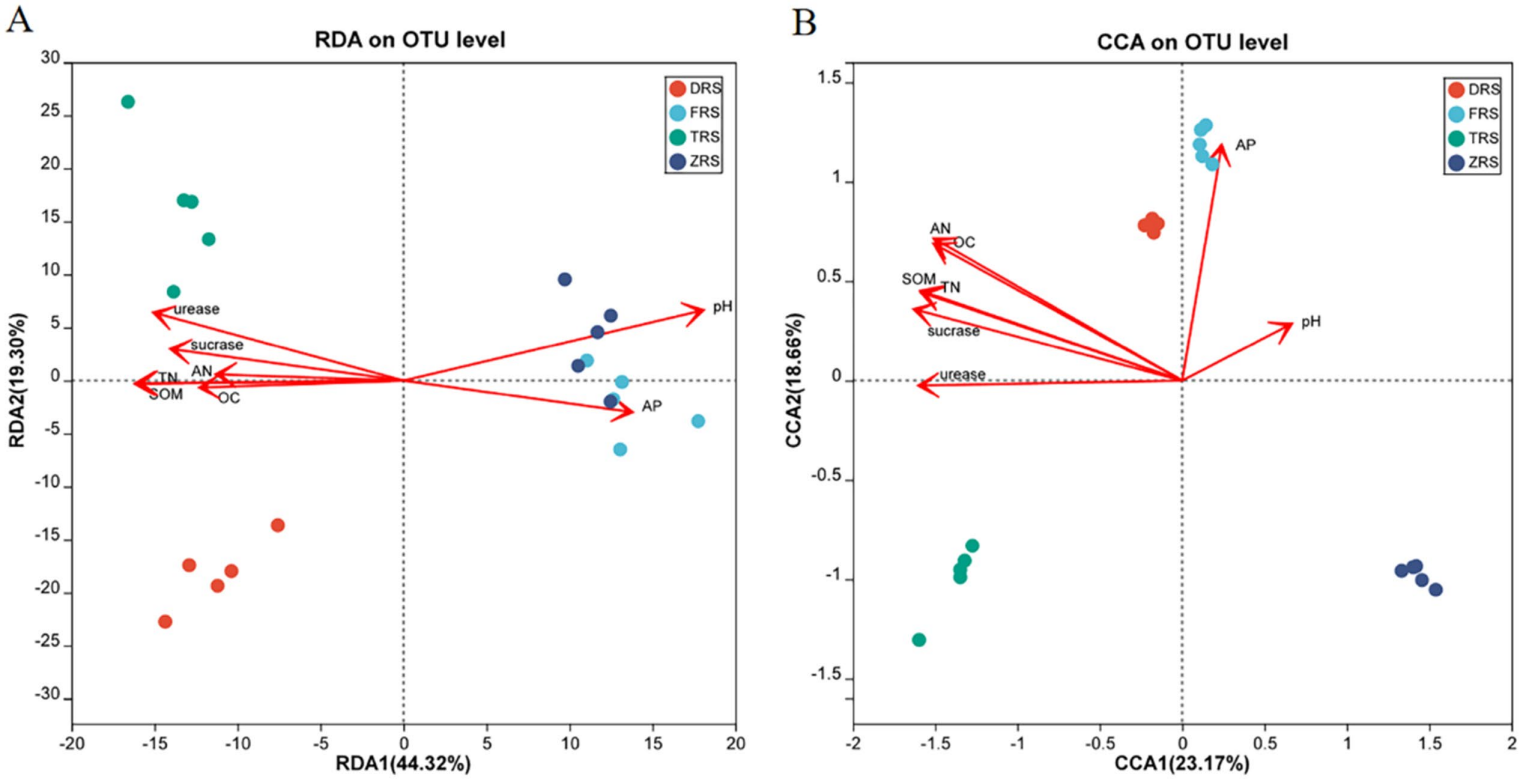
Heat map of correlations between soil bacterial phyla **(A)** and fungal phyla **(B)** and different habitats and soil physicochemical indicators.

Further analysis was conducted on the relationships between the top 10 bacterial and fungal phyla and soil physiochemical properties among habitats (Figure 12). For bacteria, soil pH was found to be significantly positively correlated with Bacteroidota and Chloroflexi, and significantly negatively correlated with Planctomycota and Verrucomicrobiota. TN, AN, SOM, and OC were found to be significantly positively correlated with Proteobacteria and Acidobacteriota, while SOM and TN were significantly negatively correlated with Chloroflexi. The contents of sucrase and urease were significantly positively correlated with Proteobacteria and Acidobacteria. For fungi, soil pH was significantly positively correlated with *Ascomycota*, *Glomeromycota*, and *Chytidiomycota*. AP was significantly positively correlated with Chytridiomycota, and significantly negatively correlated with Basidiobolomycota. TN, SOM, and sucrase and urease content were significantly negatively correlated with Chytidiomycota, Kickxellomycota, and Ascomycota.

## 4 Discussion

Rhizosphere soil is considered a highly complex and dynamic ecosystem. We observed that both the bacterial species richness and diversity were higher in the Buddha Rock and Botanical Garden habitats than in the other two habitats. Buddha Rock is a tourist attraction which is open to the public. The Botanical Garden is an artificial protection zone constructed on what was once farmland, and there is still agricultural land nearby. Our results suggest that a large number of anthropogenic activities and measures such as tillage and fertilization may have had some impact on the inter-root soil microbial community. These findings validate those of Dong et al. (2023), who found that the application of fertilizers can potentially increase microbial diversity. Huang et al. revealed that because the selection of specific microorganisms by different plant communities (e.g., legumes) promotes certain types of bacteria, this can lead to an increase in microbial diversity in agricultural fields (Huang et al., 2020). Soil bacterial diversity was higher in areas with tourist disturbances and human activities than in undisturbed areas, and Zhang’s (Zhang et al., 2019) study showed that moderate disturbance increased soil bacterial diversity.

Across all four habitats, Proteobacteria was found to have the highest relative abundance among all bacterial phyla. This bacterial phylum was also found to exhibit the highest relative abundance in many wetlands (Zhang et al., 2017; He et al., 2022). Proteobacteria are reported to play an important role in nitrogen fixation and nutrient cycling (Lv et al., 2014), suggesting that these bacteria may participate in nitrogen cycling in *A. spinulosa* protected areas. Acidobacteria were found to be the second most dominant bacterial phylum, and these bacteria play a very important role in soil material cycling and ecological environment construction. Acidobacteria were also quite common, and hold important functions in degrading plant residues and participating in iron cycling (Eichorst et al., 2011; Wang et al., 2016). The LEfSe analysis indicated that Buddha Rock contained a significantly higher relative abundance of Bacteroidetes than the other three habitats. This is likely because Buddha Rock contains several streams and many members of Bacteroidetes are found primarily in and around aquatic environments (Janssen, 2006). As mineralizers of organic carbon, many members of Bacteroidetes can increase the content of SOC, provide energy for microbial growth, and improve soil enzyme activity (Li et al., 2011). *Bacillus* and *Bradyrhizobium* found in this study are common rhizosphere growth-promoting bacteria. Among them, *Bacillus* has the ability to dissolve phosphorus, dissolve phosphorus and dissolve potassium. Among them, strains with strong ability to transform nutrients can also secrete auxin and erythromycin (Liu et al., 2018). *Bradyrhizobium* in pea rhizosphere can secrete cytokinin to promote the increase of root surface area (Dhole et al., 2023). The strains isolated from the rhizosphere of oat and wheat can produce IAA, promote the elongation of root cells and promote the growth and development of plants (Yao, 2002). IAA can increase root exudates and is an important hormone in plants. It has a direct regulatory effect on root architecture and physiological metabolism (Verbon and Liberman, 2016). IAA allows plants to absorb more nutrients and enhance the sensitivity of plants to pathogens. Therefore, it reflects that the rhizosphere bacteria of *A. spinulosa* have great potential in the development of plant growth promoting agents, which can be of great significance for promoting plant growth and development. In addition, we observed that the genus *Thermobacterium* was found at all sites except for the Botanical Garden. *Thermobacterium* decomposes organic matter and utilizes diverse carbon sources (Ye et al., 2014). The genus *Bacillus* was detected in the rhizosphere soils of Dashui Gou and the Botanical Garden. Members of the genus Bacillus are largely viewed as beneficial microorganisms which produce an array of bioactive molecules to inhibit pathogens (Ongena and Jacques, 2008). The presence of such microorganisms may serve to protect *A. spinulosa* against pathogens.

The dominant fungal phyla across all four habitats were Basidiomycota and Ascomycota. These two fungal phyla are important decomposers in soil ecosystems. Members of Ascomycota are primarily saprophytic and play a key role in the decomposition of complex organic matter (Beimforde et al., 2014), while members of Basidiomycetes degrade lignocellulose in plant residues (Frey et al., 2004). The relative abundance of Basidiomycota was significantly higher in Tiantang Gou than in the other three habitats, while the relative abundance of Ascomycota was significantly higher in the Botanical Garden. Ascomycota are primarily found during the early stages of soil fungal community succession and are considered pioneer species which quickly invade dead branches and leaves. As fungal community succession progresses, Ascomycota are gradually replaced by more competitive rot fungi and Basidiomycetes. Therefore, the higher abundance of Basidiomycetes in Tiantang Gou may reflect the relatively advanced stage of succession and mature community composition of its soil fungal community (He et al., 2014).

The correlation heatmap results revealed a positive correlation between the relative abundance of Proteobacteria and SOM, which is consistent with the soil decomposing character of Proteobacteria. The relative abundance of Acidobacteria was negatively correlated with soil pH, which is consistent with most previous studies (Zhang et al., 2014; Jones et al., 2009). However, although Acidobacteria are most commonly found under acidic soil conditions, other studies have found that the relative abundance of Acidobacteria is not significantly correlated with soil pH (Liu et al., 2015). This may be due to the presence of different subgroups of Acidobacteria, as different subgroups of acid-loving bacteria may respond differently to varied environmental factors. When analyzing the relationship between soil fungal phyla and physicochemical indicators, it was found that the relative abundance of Ascomycota was significantly negatively correlated with SOM and significantly positively correlated with soil pH. These results are similar to those reported by Du et al. (2020), who studied the soil fungal communities and physicochemical properties of spruce and birch forests.

## 5 Conclusion

Among four different habitats within the Chishui *A. spinulosa* National Nature Reserve, the bacterial phylum Proteobacteria and the fungal phylum Ascomycota were observed to be the most abundant in *A. spinulosa* rhizosphere soil. These phyla play important roles in soil material cycling and ecological environment construction. Buddha Rock and the Botanical Garden exhibited higher bacterial diversity than either Tiantang Gou or Dashui Gou, reflecting the history of human interference on these sites. Bacterial community composition and diversity were strongly influenced by soil pH, while fungal community composition and diversity were strongly influenced by AP, SOC, sucrase, and urease. The results of this study provide a scientific basis for the habitat restoration of *A. spinulosa*, and the improvement of the structure of the *A. spinulosa* rhizosphere soil microbial community. Laying a theoretical foundation for the next screening of inter-root functional flora.

## Data Availability

The datasets presented in this study can be found in online repositories. The names of the repository/repositories and accession number(s) can be found in the article/supplementary material.

## Footnotes

^1^ https://github.com/OpenGene/fastp

^2^ http://www.cbcb.umd.edu/software/flash

^3^ https://www.arb-silva.de

^4^ https://unite.ut.ee/

^5^ https://cloud.majorbio.com

^6^ https://www.R-project.org/

^7^ https://github.com/vegandevs/vegan
